# Supplementary material for: Turkish nursing students’ compliance to standard precautions during the COVID-19 pandemic
Source: PeerJ. 2023 Mar 16;11:e15056. doi: 10.7717/peerj.15056 (PMC10024895; doi:10.7717/peerj.15056)
Supplement: Supplemental Information 3 [file peerj-11-15056-s003.docx]

VERİ TOPLAMA FORMU (TR)

**Sizi Dr.Öğr.Üyesi Sevcan Topçu** ve **Dr. Öğr. Gör. Zuhal EMLEK SERT** tarafından yürütülen **“COVID-19 Pandemisinde Türk Hemşirelik Öğrencilerinin Standart Önlemlere Uyumu”** başlıklı araştırmaya davet ediyoruz. Bu araştırmaya katılıp katılmama kararını vermeden önce araştırmanın neden ve nasıl yapılacağını bilmeniz gerekmektedir. Eğer anlayamadığınız ve size açık gelmeyen yer varsa ya da daha fazla bilgi isterseniz lütfen bize sorunuz.

Bu çalışmaya katılım **gönüllülük esasına** dayanmaktadır. Çalışmaya katılmama ya da katıldıktan sonra **vazgeçme hakkına** sahipsiniz. Aşağıda verilen soruları yanıtlamanız çalışmaya katılmaya gönüllü olduğunuzu varsayacaktır. Formda bulunan soruları yanıtlarken kimsenin baskısı altında olmayınız. Bu formlardan elde **edilecek bilgiler tamamen gizli tutulacak ve araştırma amacıyla kullanılacaktır.**

**SOSYO-DEMOGRAFİK ÖZELLİKLER**

**Katılımcı No:**

1. Yaş:

2. Cinsiyet

( ) Kadın ( ) Erkek

3. Sınıf

( ) 2 ( ) 3 ( ) 4

4. Kesici-delici alet yaralanması veya kan/vücut sıvıları ile temas yaşadınız mı?

( ) Evet ( ) Hayır

**Standart Önlemler Ölçeği (CSPS)**

*Lütfen **mevcut klinik uygulamanızı** en iyi yansıtan kutuyu ✔ ile işaretleyin.

*Lütfen 20 sorunun tamamını cevaplayın.

|  | **Asla/Hiç** | **Nadiren** | **Bazen/Ara sıra** | **Her zaman** |
| --- | --- | --- | --- | --- |
| **1.** Hastadan hastaya geçişte ellerimi yıkarım. | ❏ | ❏ | ❏ | ❏ |
| **2.** El yıkarken sadece su kullanırım. | ❏ | ❏ | ❏ | ❏ |
| **3.** Ellerimde gözle görülür kirlenme yoksa, yıkamaya alternatif olarak alkollü el dezenfektanı/temizleme sıvısı kullanırım. | ❏ | ❏ | ❏ | ❏ |
| **4.** Enjeksiyon yaptıktan sonra kullandığım iğnelerin kapağını kapatırım. | ❏ | ❏ | ❏ | ❏ |
| **5.** Kesici delici özellikteki malzemeleri kullandıktan sonra kesici delici alet kutusuna atarım. | ❏ | ❏ | ❏ | ❏ |
| **6.** Kesici delici alet kutusu sadece dolduğunda atılır. | ❏ | ❏ | ❏ | ❏ |
| **7.** Kişisel Koruyucu Ekipmanı (gözlük, maske, bone, önlük vb.) özel olarak ayrılmış/belirlenmiş alanda çıkarırım. | ❏ | ❏ | ❏ | ❏ |
| **8.** Aşırı miktarda bir sıçramaya maruz kaldığımda Kişisel Koruyucu Ekipman (gözlük, maske, bone, önlük vb.) kullanmış olsam da, normalden daha uzun süren bir duş alırım. | ❏ | ❏ | ❏ | ❏ |
| **9.** Yara(larım), lezyon (larım) varsa, hastaya temas etmeden önce su geçirmez pansuman ile kapatırım. | ❏ | ❏ | ❏ | ❏ |
| **10.** Hastaların vücut sıvıları, kan ürünleri veya herhangi bir salgısına maruz kalmadan önce eldiven giyerim. | ❏ | ❏ | ❏ | ❏ |
| **11.** Hastadan hastaya geçişte eldivenlerimi değiştiririm. | ❏ | ❏ | ❏ | ❏ |
| **12.** Eldivenlerimi çıkarır çıkarmaz ellerimi uygun bir şekilde temizlerim. | ❏ | ❏ | ❏ | ❏ |
| **13.** Sıçrama, saçılma olasılığına karşı sadece yüz/cerrahi maske ya da maske ile birlikte gözlük, yüz koruyucu, önlük kullanırım. | ❏ | ❏ | ❏ | ❏ |
| **14.** Maske taktığımda ağzım ve burnum kapalı olur. | ❏ | ❏ | ❏ | ❏ |
| **15.** Bir cerrahi maskeyi ya da tek kullanımlık Kişisel Koruyucu Ekipmanı tekrar kullanırım. | ❏ | ❏ | ❏ | ❏ |
| **16.** Kan ve vücut sıvıları veya hastanın diğer salgılarına salgısına maruz kalmadan önce önlük ya da yüz koruyucu takarım. | ❏ | ❏ | ❏ | ❏ |
| **17.** Kan, vücut sıvıları, sekresyon ve salgı ile kontamine atıklar, hastanın enfeksiyon durumuna bakılmaksızın kırmızı atık torbalarına atılır. | ❏ | ❏ | ❏ | ❏ |
| **18.** Malzemeleri ve çalışma alanını kullandıktan sonra temizlerim. | ❏ | ❏ | ❏ | ❏ |
| 19. Kullandıktan sonra gözle görülür biçimde kirlenen malzemeleri temizlerken eldiven giyerim. | ❏ | ❏ | ❏ | ❏ |
| 20. Saçılan, dökülen kan ve kan ürünlerini veya diğer vücut sıvılarını hemen dezenfektanla temizlerim. | ❏ | ❏ | ❏ | ❏ |

**DATA COLLECTION FORM (EN)**

**We invite you to the research titled “Compliance with Standard Precautions among Turkish Nursing Students During The COVID-19 Pandemic” conducted by Assist.Prof.Dr..Sevcan Topçu and Dr. Lecturer Zuhal EMLEK SERT.** Before deciding whether or not to participate in this research, you need to know why and how the research will be conducted. If there is something that you do not understand and is not clear to you, or if you want more information, please ask us.

Participation in this study is on a voluntary basis. You have the right not to participate in the study or to opt out after participating. Answering the questions below will assume that you are willing to participate in the study. Do not be under pressure from anyone while answering the questions on the form. The information obtained from these forms will be kept completely confidential and will be used for research purposes.

**SOCIO-DEMOGRAPHIC CHARACTERISTICS**

**Participant Number:**

1. Age

2. Gender

( ) Women ( ) Men

3. Year of study

( ) 2 ( ) 3 ( ) 4

4. Do you experienced the experience of needle-stick injury, or contact with blood/body fluids?

( ) Yes ( ) No

**Compliance with StandardPrecautionsScale (CSPS)**

Please mark a ✔in the box that best reflects your **current clinical practice**.

Please answer all 20questions.

| **Never** | | **Seldom** | **Sometimes** | **Always** |
| --- | --- | --- | --- | --- |
| **Q1.**I wash my hands between patient contacts. | ❏ | ❏ | ❏ | ❏ |
| **Q2.**I only use water for hand washing. | ❏ | ❏ | ❏ | ❏ |
| **Q3.**I use alcoholic hand rubs as an alternative if my hands are not visibly soiled. | ❏ | ❏ | ❏ | ❏ |
| **Q4.**I recapusedneedles after giving an injection. | ❏ | ❏ | ❏ | ❏ |
| **Q5.**I put used sharp articles into sharps boxes. | ❏ | ❏ | ❏ | ❏ |
| **Q6.**The sharps box is disposed only when it is full. | ❏ | ❏ | ❏ | ❏ |
| **Q7.**I remove Personal Protective Equipment (PPE) in a designated area. | ❏ | ❏ | ❏ | ❏ |
| **Q8.**I take a shower in case ofextensive splashing evenafter I have put onPersonal Protective Equipment(PPE). | ❏ | ❏ | ❏ | ❏ |
| **Q9.**I cover my wound(s) or lesion(s) with waterproof dressing before patient contacts. | ❏ | ❏ | ❏ | ❏ |
| **Q10.**I wear gloves when I am exposed to body fluids, blood products,and any excretion of patients. | ❏ | ❏ | ❏ | ❏ |
| **Q11.**I change gloves between patient contacts. | ❏ | ❏ | ❏ | ❏ |
| **Q12.**I decontaminate my hands immediately after removal of gloves. | ❏ | ❏ | ❏ | ❏ |
| **Q13.**I wear a surgical maskalone or in combination withgoggles, face shield and apron whenever there is a possibility of a splash or splatter. | ❏ | ❏ | ❏ | ❏ |
| **Q14.**My mouth and nose are coveredwhen Iwear a mask. | ❏ | ❏ | ❏ | ❏ |
| **Q15.**I reuse a surgical mask or disposable Personal Protective Equipment(PPE). | ❏ | ❏ | ❏ | ❏ |
| **Q16.**I wear a gown or apronwhen exposed toblood, body fluids or any patient excretions. | ❏ | ❏ | ❏ | ❏ |
| **Q17.**Waste contaminated with blood, body fluids, secretion and excretion isplaced in red plastic bagsirrespective of the patient’s infection status. | ❏ | ❏ | ❏ | ❏ |
| **Q18.**I decontaminate surfaces and equipment after use. | ❏ | ❏ | ❏ | ❏ |
| **Q19.**I wear gloves to decontaminate used equipment with visible soils. | ❏ | ❏ | ❏ | ❏ |
| **Q20.**I clean up spillage of blood or other body fluidsimmediately with disinfectants. | ❏ | ❏ | ❏ | ❏ |
